# The value of local dexmedetomidine as an adjuvant to ultrasound-guided wide awake local anesthesia no tourniquet (WALANT) in flexor tendon repair surgeries: a randomized controlled trial

**DOI:** 10.1186/s12871-024-02504-x

**Published:** 2024-03-27

**Authors:** Mahmoud Mohammed Alseoudy, Elsayed Mohamed Abdelkarime, Khaled Nour, May Elsherbiny Badr

**Affiliations:** 1 Department of Anesthesia and Surgical Intensive Care, Faculty of Medicine, Mansoura University, Abdelsalam Aref St., Mansoura City, El-Dakahliya Governorate Egypt; 2 Department of Orthopedic Surgery, Faculty of Medicine, Mansoura University, Mansoura, Egypt

**Keywords:** WALANT, Dexmedetomidine, Morphine, Flexor tendon repair, Analgesia, Ultrasound

## Abstract

**Background:**

The Wide-Awake Local Anesthesia No Tourniquet (WALANT) technique allows intraoperative motor assessment of tendon repair integrity of the hand compared with general anesthesia or brachial plexus block. No studies have tested the effect of adding dexmedetomidine to lidocaine on the analgesic properties of the WALANT technique, which is the aim of our study.

**Methods:**

A total of 128 patients aged more than 18 years were scheduled for surgical flexor tendon injury repair using WALANT technique. Patients were divided into two equal groups. Ultrasound-guided subcutaneous injection of lidocaine 1% with dexmedetomidine (1 µg/kg), Group D, or without dexmedetomidine, Group C, was performed at four points: proximal to the wrist joint, the distal forearm, palm region, and proximal phalanges. The primary outcome was total morphine consumption throughout the first postoperative day. Secondary outcomes included number of patients requiring rescue analgesia, time to first analgesic request, and pain score.

**Results:**

Total morphine consumption was significantly (*P* < 0.001) lower in group D (2.66 ± 0.998) than in group C (3.66 ± 1.144) mg. Number of patients requiring rescue analgesia was significantly (*P* < 0.001) lower in group D (54.7% (35)) than group C (100.0% (64)). The time for first request for analgesia was significantly (*P* < 0.001) longer in group D (11.31 ± 6.944) than in group C (5.91 ± 4.839) h. Pain score was significantly higher in group C than D at three (*P* < 0.001), and six (*P* = 0.001) hours (*P* = 0.001) postoperatively.

**Conclusion:**

Dexmedetomidine significantly improves the analgesic quality of WALANT when added to lidocaine with less opioid consumption.

**Trial registration:**

(ID: PACTR202203906027106; Date: 31/07/2023).

## Introduction

Successful management of flexor tendon injuries depends mainly on surgical repair and post-operative rehabilitation with the aim of preventing tendon gapping or rupture, minimizing post-operative adhesions, and optimizing functional outcomes [1–3]. During surgeries for tendon repair, it is recommended to assess the strength of the repaired bowstringing [4] and execute a digital extension-flexion test [5]. The active tests could not be reliably performed if the patient was under general anesthesia, sedation, brachial plexus block, or local anesthesia with sedation. Without this active testing, the surgeon is less confident about the success of repair, with a greater risk of gapping or rupture. [2].

Over the previous two decades, the “Wide-Awake Local Anesthesia No Tourniquet (WALANT) technique” has gained popularity as a local anesthetic technique for hand surgeries that involves preoperative installation of lidocaine and epinephrine in the operative field to provide anesthesia and hemostasis [6, 7]. WALANT has multiple advantages, such as omitting general anesthesia with its complications, no need for patient fasting, possible patient discharge on the same operation day, and low financial costs. In addition, WALANT eliminates the need for a tourniquet and allows for performing tendon repair in an awake patient, which enables the surgeon to test the repair for gapping or impingement on the pulley [8–12].

Despite the previous advantages, a short-acting local anesthetic agent is usually used in the WALANT procedure, which could lead to inadequate postoperative analgesia. Dexmedetomidine is a highly selective alpha-2 adrenergic receptor agonist that is widely used in the field of anesthesia and intensive care for sedation, analgesia, and anxiolysis [13, 14]. It is also used as an adjuvant to local anesthetic agents because it prolongs and enhances their analgesic action [15, 16].

To the best of our knowledge, there is an obvious paucity of studies evaluating the beneficial effect of dexmedetomidine on patients undergoing surgery under WALANT. We hypothesized that the addition of dexmedetomidine to lidocaine/epinephrine solution would improve the analgesic properties of WALANT in patients undergoing flexor tendon repair.

## Patients & methods

This double-blind prospective randomized controlled study was conducted in the orthopedic operating rooms of the main Mansoura University Hospital and emergency hospital, after gaining approval from the local scientific committee and Institutional Review Board (R.22.03.1648) of our medical school. The study was conducted between April 2022 and November 2023. This study was registered in the Pan African Clinical Trials Registry (ID: PACTR202203906027106; Date: 31/07/2023). Written informed consent was obtained from each patient before surgery. This study was conducted in accordance with the ethical principles of the Declaration of Helsinki (2013) and was consistent with good clinical practice.

The study was conducted on 128 patients. Patients of both sexes, aged more than 18 years, with a flexor tendon injury in the hand region, scheduled for surgical intervention under WALANT, and classified as class I or II according to the American Society of Anesthesiologists (ASA) physical status score, were included in the study.

Patients were excluded in the event of refusal of the anesthesia technique, diagnosis of finger tendon lesions, concomitant bony fractures or requiring digital nerve repair, bleeding diathesis, ASA class III or higher, history of drug addiction, and allergy to any of the study medications.

Patients were divided into two equal groups (each of 64 patients); Group D included patients who received WALANT with dexmedetomidine, and Group C included patients who received WALANT only by computer randomization code. Allocation concealment was done using a closed envelope system and by an anesthetist who was not involved in the study. The attending anesthesiologists and data collectors were blinded to the drug being administered.

In the ward, all patients were assessed via history-taking, clinical examination, and routine preoperative laboratory investigations. The WALANT procedure was explained to all participants. They were also taught how to express their pain using the numerical rating scale (NRS), with 0 for no pain and 10 for the worst pain ever. They were instructed to ask for analgesia only if the NRS was more than 3 at rest.

In the operating room, patients were monitored with a 5-lead ECG, pulse oximetery, and noninvasive blood pressure. Along with the basal pulse and mean arterial blood pressure (MAP) readings, these variables were measured at 10, 20, 30, 45, and 60 min after WALANT application. For both groups, injection was performed 30 min before the surgery. We used a solution containing 1% lidocaine and 1:100,000 epinephrine in both groups but adding 1 µg/kg dexmedetomidine for Group D. We strictly adhere to the safe limit of 7 mg/kg for lidocaine with epinephrine. Local anesthesia was injected into the subcutaneous tissues in a tumescent fashion. The block was performed by ultrasound-guided, either out-of-plane or in-plane, subcutaneous injection at four points. We started with 2 ml of the injectate at a point 2 cm proximal to the wrist joint. This was followed by an 8-ml injection under the skin of the distal forearm. Subsequently, approximately 20 ml of the same injectate was installed into the palm near the planned area of incision, 10 ml over the carpal tunnel region, and 10 ml over the distal palm region. Finger injection with 2 ml in the center of each phalanx was performed if exposure was needed. The attending anesthetist assessed the block effectiveness by pinprick at the planned surgical incision and then the surgery was allowed. The duration of sensory blockade was assessed by the pinprick method every 15 min after end of surgery by the attending anesthetist who was blind to the study group. In case of WALANT failure (anesthesia is insufficient and patients claim of pain during surgery), general anesthesia would be a rescue therapy. After the operation, all patients were transferred to the internal ward, where they were monitored by a dedicated monitor for the occurrence of hypotension, bradycardia, or oxygen desaturation. NRS was evaluated by an anesthetist who was blind to the study group. NRS was performed 5 times at 1, 3, 6, 12, and 24 h after the surgery. All patients received IV paracetamol (1gm every 8 h) and ketorolac (30 mg every 12 h) as boluses on the due times, given initially once patient had reached the recovery room. If the patient reported NRS of more than three, IV morphine was given (2 mg) as a bolus given slowly over one min. The primary objective of the study was to determine the total morphine consumption throughout the first postoperative day. The secondary objectives were hemodynamic parameters, pain score, duration of sensory blockade, time to first analgesic request, number of patients requiring rescue morphine analgesia, and patient satisfaction. Patients’ satisfaction with postoperative analgesia was assessed on a three-point verbal rating scale (1 = dissatisfied since they had severe pain, 2 = satisfied as there was minimal pain only;, 3 = extremely satisfied as there was no pain).

### Sample size calculation and statistical analysis

The sample size was computed using the PASS software tool, with the primary outcome being post-operative analgesic intake on the first day after surgery. The null hypothesis was that there was no difference in post-operative analgesic consumption between the two treatment modalities. Based on previous literature research, no previous studies have compared both modalities regarding post-operative analgesic consumption. A sample size of 64 patients in each group is needed to achieve 80% power and detect an effect size of 0.5 (moderate effect size) in the current study using a two-sided two-sample equal-variance t-test with a significance level of 5%. IBM’s SPSS statistics (Statistical Package for the Social Sciences) for Windows (version 24) was used for statistical analysis of the collected data. The Shapiro–Wilk test was used to check the normality of the data distribution. Normally distributed continuous variables were expressed as mean ± SD while categorical variables and abnormally distributed continuous variables were expressed as a median and interquartile range or as numbers and percentages (as appropriate). Student’s t-test and Mann–Whitney U were used for normally and abnormally distributed continuous data respectively. Chi-square test was used for categorical data using the crosstab function. All tests were conducted with a 95% confidence interval. If needed, bivariate correlations were assessed using Pearson’s or Spearman’s correlation coefficients depending on the nature of the data. *P* (probability) value < 0.05 was considered statistically significant.

## Results

**Fig. 1:**
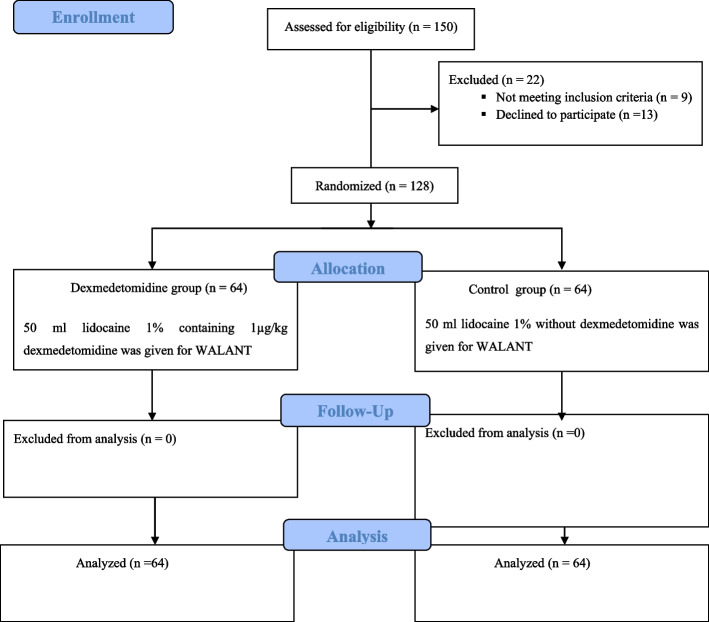
Flowchart demonstrating recruitment of patients in both study and control groups. WALANT: Wide Awake Local Anesthesia No Tourniquet

We enrolled 128 patients in our study, with 64 patients in each group (Fig. 1). There was no statistically significant difference in demographic data, medical history, and duration of surgery between the two groups (Table 1). Patients’ hemodynamic parameters (heart rate and mean arterial blood pressure) were comparable (*P* > 0.05) at all times of measurement between group D and C (Tables 2 and 3).

**Table 1:** Demographic characteristics, ASA class, medical history, and duration of operation in group D and C

	**GroupD (*n* = 64)**	**GroupC (*n* = 64)**	**95% CI**	***P***
**Age (years)**	43.75 ± 10.092	42.44 ± 11.660	-2.50, 5.13	0.497
**Gender (male/ female)**	33/31	40/24	-0.06, 0.28	0.211
**BMI (kg/m**^**2**^**)**	31.14 ± 4.708	31.25 ± 3.358	-1.54, 1.32	0.884
**ASA (I/ II)**	51/13	52/12	-0.12, 0.15	0.824
**History of diabetes(%,N)**	10.9% (7)	9.4% (6)	-0.12, 0.09	0.770
**History of hypertension(%,N)**	7.8% (5)	10.9% (7)	-0.07, 0.13	0.544
**Duration of surgery (minutes)**	64.69 ± 13.566	68.44 ± 12.404	-8.30, 0.80	0.105

Data are expressed as mean ± SD, ratio or number and percent

*group D* Patients injected with dexmedetomidine plus lidocaine/ epinephrine, *group C* Patients injected only with lidocaine/ epinephrine, *BMI* Body mass index, *ASA* American society of anesthesiologists

**Table 2:** Heart rate changes in the two study groups

**Heart rate** (beat/min)	**Group D (*n* = 64)**	**Group C (*n* = 64)**	**95% CI**	***P***
**Basal**	76.36 ± 9.597	73.36 ± 8.456	-0.16, 6.16	0.063
**10 min**	80.28 ± 10.332	78.11 ± 9.600	-1.32, 5.66	0.220
**20 min**	81.11 ± 11.322	79.16 ± 10.910	-1.94, 5.84	0.322
**30 min**	79.45 ± 9.827	79.41 ± 10.593	-3.53, 3.62	0.979
**45 min**	78.52 ± 8.972	78.34 ± 10.993	-4.08, 4.44	0.932
**60 min**	77.36 ± 10.135	80.47 ± 12.470	-9.61, 3.41	0.343

Data are presented as mean ± standard deviation

*Group D* Patients injected with dexmedetomidine plus lidocaine/ epinephrine, *group C* Patients injected only with lidocaine/ epinephrine

**Table 3:** MAP (mmHg) follow-up in the two study groups

**MAP** (mmHg)	**Group D (*n* = 64)**	**Group C (*n* = 64)**	**95% CI**	***P***
**Basal**	95.89 ± 6.515	94.98 ± 6.975	-1.45, 3.27	0.449
**10 min**	94.58 ± 8.587	92.83 ± 10.542	-1.62, 5.12	0.305
**20 min**	96.19 ± 9.053	92.64 ± 11.707	-0.11, 7.21	0.057
**30 min**	95.64 ± 9.918	93.08 ± 11.871	-1.26, 6.39	0.187
**45 min**	95.74 ± 9.495	92.49 ± 11.267	-1.17, 7.67	0.148
**60 min**	96.55 ± 8.319	91.63 ± 10.730	-0.61, 10.43	0.080

Data are presented as mean ± standard deviation

*Group D* Patients injected with dexmedetomidine plus lidocaine/ epinephrine), *group C* Patients injected only with lidocaine/ epinephrine

The duration of the sensory block (hour) was significantly longer (*P* < 0.001) in group D (7.22 ± 3.195) than in group C (3.19 ± 0.732). The numerical rating scale was comparable between the two groups except at 3 and 6 h postoperatively with *P* values of < 0.001 and 0.001, respectively. The number of patients who needed rescue analgesia was significantly lower (*P* < 0.001) in group D (54.7% (35)) than in group C (100.0% (64)). The total amount of morphine (mg) was significantly lower (*P* < 0.001) in group D (2.66 ± 0.998) than in group C (3.66 ± 1.144). Our study also showed a significant difference between groups D and C regarding the time for their first request for analgesia (hour) and patient satisfaction, with a *P* value of < 0.001 (Table 4). There were no reported cases of WALANT failure or postoperative hypotension, bradycardia, or oxygen desaturation in either group.

**Table 4:** Sensory block duration and postoperative analgesic profile in the two study groups

		**Group D (*n= 64)***	**Group C (n= 64)**	**95% CI**	***P***
**Sensory block duration (Hours)**		7.22 ± 3.195	3.19 ± 0.732	3.22, 4.84	˂ 0.001 *
**NRS**	**One hour**	0.30 ± 0.460	0.33 ± 0.473	-0.19, 0.13	0.706
	**Three hours**	0.53 ± 0.534	3.28 ± 1.215	-3.08, - 2.42	˂ 0.001*
	**Six hours**	2.75 ± 1.222	3.44 ± 1.022	-1.08, -0.29	0.001*
	**12 hours**	2.56 ± 1.022	2.91 ± 0.955	-0.69, 0.00	0.051
	**24 hours**	2.59 ± 0.904	2.64 ± 1.226	-0.42, 0.33	0.806
**Number of patients requiring rescue**					
**Analgesia (%,N)**		54.7% (35)	100.0% (64)	0.33, 0.57	˂ 0.001*
**First request for analgesia (hours) **		11.31 ± 6.944	5.91 ± 4.839	3.04, 7.77	˂ 0.001*
**Morphine (mg)**		2.66 ± 0.998	3.66 ± 1.144	-1.46, -0.54	˂ 0.001*
**Patient satisfaction**	**Dissatisfied**	6.3% (4)	46.9% (30)		
	**Satisfied**	40.6% (26)	28.1% (18)	-	˂ 0.001*
	**Extremely satisfied**	53.1% (34)	25.0% (16)		

Data are expressed as mean ±SD or number and percent. Group D (patients injected with dexmedetomidine plus lidocaine/ epinephrine), group C (patients injected only with lidocaine/ epinephrine), NRS (numerical rating scale), *P < 0.05 is statistically significant

## Discussion

To the best of our knowledge, this is the first randomized controlled trial evaluating the value of adding dexmedetomidine to lidocaine/epinephrine solution in the analgesic properties of WALANT in patients undergoing flexor tendon repair. Our results showed that the duration of sensory block and the time for the first request for analgesia were significantly longer in the dexmedetomidine group than in the control group. The total morphine consumption was significantly lower in the dexmedetomidine group than in the control group, and the number of patients requiring rescue analgesia was significantly lower in the dexmedetomidine group than in the control group. The pain score was significantly higher in the control group than in the dexmedetomidine group at 3 and 6 h postoperatively, and patient satisfaction was higher in the dexmedetomidine group than in the control group.

Although the FDA-approved indications for dexmedetomidine are sedation of intubated and mechanically ventilated patients in the intensive care unit and peri-procedural sedation of non-intubated patients, a bulk of published data about its off-label effective and safe use as an adjuvant to local anesthetic for peripheral nerve block prolongation [17] encouraged us to use dexmedetomidine for WALANT.

In the current study, adding dexmedetomidine to WALANT was associated with a significant increase in the duration of sensory block (*p* < 0.001). Dexmedetomidine can induce a blockade of the hyperpolarization-activated cation current [18], which could explain its effect. In the same context, multiple studies have confirmed that the same adjuvant medication was associated with a significant prolongation of the sensory block duration when injected with a local anesthetic drug, regardless of the administration route (caudal, epidural, or spinal) [15, 19–21]. It also enhances the peripheral and central blockade of local anesthetic agents [22].

In our study, administration of dexmedetomidine to the WALANT injectate was associated with a significant decrease in pain scores at 3- and 6-h readings. The peripheral sensory neural block of dexmedetomidine could be explained by its actions on adrenergic alpha-2A receptors [22]. In addition, it prevents substance P release from the dorsal root neuron in the pain pathways [23]. Other researchers have reported a significant decline in local proinflammatory cytokines after the administration of dexmedetomidine with local anesthetics [24]. Furthermore, local or systemic administration of the same drug is associated with a significant improvement in sleep quality, which has a positive impact on pain sensation. Sleep disorders aggravate the intensity of post-operative pain [25].

Our findings showed that dexmedetomidine administration was associated with a significant prolongation of the first analgesic request, a significant decrease in patients requiring rescue analgesia, and a significant decline in post-operative morphine consumption. The previous findings could be explained by the prolonged sensory block and better pain scores in the dexmedetomidine group than in the control group., as mentioned before.

Our findings showed a significant improvement in patient satisfaction with dexmedetomidine. Of course, this is a reasonable finding secondary to the better analgesic profile in that group, which had a beneficial impact on patient satisfaction. Patients expressing pain commonly report lower satisfaction levels than their counterparts without pain [26].

As dexmedetomidine has a sympatholytic action, it may lead to bradycardia and hypotension through its action on presynaptic alpha receptors, which in turn, will lead to a decrease in sympathetic outflow [27]. This was not noticed in our study, and this could be explained by its local administration. In addition, adding epinephrine to the injectate is associated with local vasoconstriction, which leads to a decrease in systemic absorption when the drug is locally injected [28].

The existing literature shows a clear paucity of studies addressing the use of dexmedetomidine for WALANT, and this constitutes a strong point in favour of our investigation. The improved quality of WALANT by adding dexmedetomidine to lidocaine, according to our results, makes WALANT a suitable alternative to general anesthesia or brachial plexus block for hand surgery.

The current investigation has some limitations. This study was conducted in a single center, and the collected patient sample was relatively small. Unavailable data regarding the degree of surgical aggression, severity of injury before surgery, preoperative pain score, and time after injury before surgery, which may affect the analgesic requirements, are limitations of our study.

## Conclusion

Based on the previous findings, adding local dexmedetomidine as an adjuvant to lidocaine in WALANT is associated with a significant improvement in the analgesic profile of this local anesthetic modality. Its use should be encouraged in this setting to achieve optimum post-operative analgesic outcomes.

## Data Availability

The datasets used and analyzed during the current study are available from the corresponding author on reasonable request.

## Abbreviations

WALANT: Wide Awake Local Anesthesia No Tourniquet
ASA: American Society of Anesthesiologists
h: Hour
MAP: Mean arterial blood pressure
SPSS: Statistical Package for the Social Sciences
NRS: Numerical rating scale
